# Pharmacological evaluation of *Adenostemma lavenia* acetone extract in Swiss Albino mice: Analgesic, anti‐inflammatory, and thrombolytic insights from in vivo, in vitro, density functional theory, and molecular docking studies

**DOI:** 10.1002/ame2.70200

**Published:** 2026-04-12

**Authors:** Nusrat Jahan Moon, Mahathir Mohammad, Md. Jahirul Islam Mamun, Fahmina Binty Azim Nova, Nazmul Hasan Eshaque, Md. Hossain Rasel, Zobayed Islam, Md. Mahmudul Hasan, Md. Liakot Ali, Md. Tanvir Chowdhury, S. M. Moazzem Hossen

**Affiliations:** ^1^ Department of Pharmacy Daffodil International University Dhaka Bangladesh; ^2^ Department of Chemistry Chittagong University of Engineering & Technology Chittagong Bangladesh; ^3^ Department of Pharmacy, Faculty of Biological Sciences University of Chittagong Chittagong Bangladesh; ^4^ Department of Pharmacy University of Science and Technology Chittagong (USTC) Chittagong Bangladesh

**Keywords:** *Adenostemma lavenia*, ADME/T, analgesic, DFT, HOMO–LUMO, in silico

## Abstract

**Background:**

*Adenostemma lavenia,* a plant long used in traditional medicine to manage pain, inflammation, and circulatory disorders, has not been thoroughly validated using modern scientific methods. This study presents the first comprehensive investigation into the analgesic, anti‐inflammatory, and thrombolytic potentials of the acetone extract of *A. lavenia* leaves (AEAL), employing an integrated strategy that combines in vivo, in vitro, and in silico methodologies.

**Methods:**

The analgesic effects of the extract AEAL were evaluated in mice (five mice per group) using acetic acid‐induced writhing, formalin‐induced pain, and hot plate tests. Anti‐inflammatory activity was assessed through xylene‐induced ear edema in mice, heat‐induced hemolysis of human red blood cells (HRBC), and protein denaturation assays. In vitro clot lysis was performed in triplicate to determine the thrombolytic activity of AEAL and a standard streptokinase. Molecular docking, density functional theory (DFT), and ADME/T profiling were conducted to identify and evaluate potential bioactive compounds.

**Results:**

AEAL demonstrated significant, dose‐dependent analgesic effects, reducing writhing responses by 53.74% (at 400 mg/kg), and inhibiting formalin‐induced pain by up to 65.76% (*p* < 0.01). It also showed potent anti‐inflammatory activity, with edema inhibition of 55.17% at a 400 mg/kg dose (*p* < 0.001), HRBC membrane stabilization (77.19%), and 85.96% suppression of protein denaturation. In thrombolytic testing, AEAL significantly (*p* < 0.001) achieved 68.1% clot dissolution compared with the control group. In silico analysis revealed that sesquiphellandrene strongly bound to COX‐2 (−7.7 kcal/mol) and tissue plasminogen activator (tPA) (−6.9 kcal/mol), whereas derivatives of 2,3‐hexadienoic acid exhibited notable COX‐2 inhibition (−7.4 kcal/mol). ADMET predictions indicated favorable drug‐like properties. In the DFT analysis, sesquiphellandrene and 2,3‐hexadienoic acid, 2‐methyl‐4‐phenyl‐, methyl ester exhibited a narrow HOMO–LUMO energy gap, indicating high chemical reactivity and a greater propensity for electron‐transfer processes.

**Conclusions:**

The AEAL exhibits robust analgesic, anti‐inflammatory, and thrombolytic activities, corroborated by computational analysis of its phytoconstituents. These results validate its ethnopharmacological use and highlight sesquiphellandrene as a promising candidate for future therapeutic development. Further isolation, mechanistic studies, and preclinical evaluation of sesquiphellandrene are warranted to harness its full pharmacological potential.

## Graphical abstract

*Adenostemma lavenia*, a traditionally used medicinal plant, has been employed to manage pain, inflammation, and circulatory issues. This study investigates the analgesic, anti‐inflammatory, and thrombolytic potentials of the acetone extract of *A. lavenia* leaves (AEAL) using in vivo, in vitro, and in silico methods.

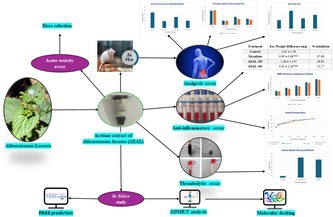

## 1 INTRODUCTION

Pain and inflammation are immune‐mediated responses that manifest as swelling, heat, immobility, redness, and discomfort when the body encounters injury, harmful stimuli, or pathogens. Pain was initially defined as an unpleasant sensory and emotional experience linked to actual or potential tissue damage or described in relation to such damage. The primary features of inflammation, vasodilation (leading to redness, warmth, and swelling), pain, and tissue reaction are largely driven by the synthesis and biological actions of prostaglandins, particularly PGE2, at the site of injury.^1^, ^2^ Over‐the‐counter options for managing pain and inflammation include drugs such as acetaminophen, diclofenac, ketorolac, and certain opioids. Widely used analgesics comprise agents like aspirin, codeine, and morphine. Nonsteroidal anti‐inflammatory drugs (NSAIDs), including aspirin, ibuprofen, naproxen, and indomethacin, are among the most frequently prescribed anti‐inflammatory agents. Their therapeutic effect arises from the inhibition of cyclooxygenase (COX) enzymes, which suppresses prostaglandin (PG) synthesis, thereby alleviating pain and inflammation.^3^, ^4^, ^5^ These symptoms are commonly treated with steroidal and non‐steroidal anti‐inflammatory drugs (NSAIDs); however, prolonged use of NSAIDs may cause gastrointestinal bleeding.^5^, ^6^, ^7^ Moreover, opioid‐based pain relievers are linked to numerous adverse effects and toxicities, such as dependency and respiratory complications. In light of these limitations, ongoing exploration of medicinal plants and their bioactive constituents is essential, as many are traditionally reported to possess promising efficacy in alleviating pain and inflammation.^8^

Thrombosis involves abnormal blood clot formation due to excessive fibrin deposition, triggered by the coagulation cascade. Fibrin is normally broken down by plasmin, generated from plasminogen by activators such as tPA or streptokinase. Thrombolytic agents promote this fibrinolysis, dissolving clots. Thus, in vitro thrombolytic testing—measuring clot lysis—helps identify compounds that may treat thrombotic disorders like stroke or heart attack.^9^, ^10^ Thrombosis within the circulatory system can lead to serious, life‐threatening conditions such as thromboembolism, stroke, ischemia, and myocardial infarction.^11^ Thrombosis has emerged as a leading cause of mortality in recent times. Currently available thrombolytic agents, such as urokinase, streptokinase, and tissue plasminogen activators, are widely used but are often associated with significant adverse effects.^12^ Hence, there is a growing need for novel or alternative therapeutic agents to manage thrombosis. Plant‐derived compounds, particularly those rich in phenolic constituents, have been reported to exhibit promising thrombolytic activity.^13^

Computer‐simulated screening, particularly through molecular docking and density functional theory (DFT) calculations, provides a robust framework for elucidating the pharmacological and physicochemical behavior of phytochemicals. Molecular docking enables the prediction of how natural ligands interact with target proteins by identifying binding sites and simulating key physicochemical interactions—such as hydrogen bonds and hydrophobic contacts—within the 3D protein structure, thereby guiding rational drug design.^14^ Complementing this, DFT analysis offers quantum‐level insights into molecular reactivity by computing electronic parameters—including HOMO (*E*_HOMO_) and LUMO (*E*_LUMO_) energies, energy gap (Δ*ε*), electronegativity (*χ*), hardness (*η*), softness (*S*), electrophilicity index (*ω*), and electron charge transfer (Δ*N*)—which collectively describe a molecule's stability, reactivity, and interaction potential. In this study, these DFT‐derived descriptors were used not only to assess biological activity but also to evaluate the adsorption mechanism of phytoconstituents, demonstrating the dual applicability of these computational tools in both drug discovery and corrosion inhibition research.^15^

Scientific research into traditional and plant‐based therapeutics plays a pivotal role in standardizing the quality and safety of clinical drugs. This paves the way for the development of sustainable, safe, and scientifically persuasive therapeutic agents that meet modern regulatory and clinical expectations.^16^ According to the World Health Organization, 70%–80% of the population in developing countries relies on complementary medicine for their primary healthcare, with herbal remedies being the main source.^17^ In Bangladesh, a significant portion of the population, particularly in rural areas, relies on medicinal plants as their primary source of treatment due to limited access to healthcare facilities. Consequently, there is growing interest in isolating bioactive compounds from these plants and assessing their pharmacological properties.^18^ *Adenostemma lavenia* is a perennial herb belonging to the Asteraceae family and is widely distributed across tropical regions of Asia.^19^ *A. lavenia* has traditionally been employed across Asia and the Pacific Islands for treating a variety of conditions, including pneumonia, fever, hepatitis, lung congestion, pulmonary embolism, and edema.^20^ The plant has also been reported to be rich in flavonoids, alkaloids, terpenoids, and essential oils, all of which possess potential antioxidant properties.^21^

Despite its extensive use in traditional medicine for the treatment of pain, inflammation, and circulatory disorders, the pharmacological potential of the acetone extract of *A. lavenia* leaves (AEAL) as an analgesic, anti‐inflammatory, and thrombolytic agent remains underexplored. Given the global burden of conditions such as chronic pain, inflammatory diseases, and thrombotic disorders—and the urgent need for safer, effective, and natural therapeutic alternatives—this study was designed to comprehensively evaluate AEAL using a multifaceted approach. Integrating in vivo, in vitro, DFT, and molecular docking analyses, this research aims to provide scientific validation for AEAL's traditional uses and identify its bioactive constituents with therapeutic promise.

## 2 MATERIALS AND METHODS

### 2.1 Plant collection and identification

*A. lavenia* leaves were collected from the Chattogram Hill Tracts in March 2024. The authenticity of the plant's leaves was verified by Mr. Md. Syedul Alam, a taxonomist at the Bangladesh Forest Research Institute (BFRI), Chattogram. A voucher specimen was preserved for future reference (accession no.: BFRIH‐351/SC).

### 2.2 Preparation of the plant extract

The extraction was carried out at room temperature by soaking approximately 550 g of powdered plant material in 2 L of acetone in an Erlenmeyer flask. To ensure efficient extraction, the mixture was left to stand for 15 days with periodic shaking and stirring. Following this maceration period, the solution was filtered through Whatman No. 1 filter paper, and the resulting filtrate was concentrated under reduced pressure using a rotary evaporator. This process yielded 18 g of crude acetone extract, which was stored at 4 °C to maintain stability and preserve bioactivity for subsequent experimental analyses.

### 2.3 Chemicals and reagents

Acetic acid, xylene, formalin, methanol, and Tween‐80 were obtained from Sigma‐Aldrich (St. Louis, MO, USA). NaCl, phosphate buffer, EDTA, diclofenac sodium, and Streptokinase (STK) vials (1 500 000 IU) were procured from Beximco Pharmaceuticals Ltd. All chemicals used in the study were of analytical grade.

### 2.4 Experimental animals

Swiss Albino mice (4–5 weeks old, 25–30 g) were procured from BCSIR, Chittagong, and acclimatized for 1 week before the experiments. They were maintained under controlled conditions (25 ± 1 °C, 55%–65% relative humidity, 12‐h light–dark cycle) in the Department of Pharmacy at the University of Chittagong, Bangladesh. The animals were housed in sterile polypropylene cages with access to clean water and a standard diet and were subjected to 12‐h food deprivation before and during the experimental procedures.^22^

### 2.5 Acute toxicity assay

The mice were kept under controlled conditions (25°C, 12‐h light/dark cycle) and were marked on the tail for identification, with a 1‐week acclimation period. In accordance with OECD guidelines, each group received specific oral treatments with extract doses ranging from 100, 500, 1000, 2000, and 4000 mg/kg body weight. After oral administration, the mice were fasted for an additional 3–4 h to ensure proper absorption and minimize food interference. The animals were then closely monitored for signs of acute toxicity or behavioral changes, including alterations in the eyes, mucous membranes, skin, and fur condition, as well as circulatory and respiratory rates, and the functions of the autonomic and central nervous systems. This observational protocol was conducted to assess the safety profile and potential adverse effects of the extract at varying dose levels. The therapeutic dose was determined as one‐tenth of the median lethal dose (LD_50_) using Karber's arithmetic method^23^ and aligned with the Hodge and Sterner toxicity classification (LD_50_ >2.0 g/kg).^24^ The LD_50_ value was obtained using the following formula:

$$LD_{50}=LD_{100}-\sum \left(a\times b\right)/n$$

In this context, *n* denotes the total number of animals in each group, *a* is the dose increment between consecutive treatments with the extract AEAL, *b* denotes the average number of deaths between two successive doses, and LD_100_ denotes the dose that induces 100% mortality in the test animals.

### 2.6 Experimental design

The mice were randomly divided into four groups (*n* = 5 per group) to evaluate the analgesic and anti‐inflammatory activities of the AEAL. Group I (negative control) received only the vehicle (e.g., 1% Tween‐80 in saline) to establish baseline responses, whereas Group II (positive control) was administered diclofenac sodium, the standard reference drug for both analgesic and anti‐inflammatory assessments. Group III received AEAL at a dose of 200 mg/kg body weight, and Group IV received a higher dose of 400 mg/kg body weight, enabling the evaluation of dose‐dependent effects. This experimental design facilitated a comprehensive comparison of AEAL's efficacy relative to both untreated and standard drug–treated controls across multiple pharmacological endpoints.

### 2.7 Analgesic assay

#### 2.7.1 Acetic acid‐induced writhing tests

This assay was conducted to evaluate the analgesic potential of the plant extracts.^25^ The mice were grouped and treated as described in Section 2.6. After oral administration of the control, standard drug, or test extract, pain was induced by intraperitoneal injection of 0.7% acetic acid (1 mL) for 40 min. Five minutes after the acetic acid injection, the cumulative number of writhing responses for each animal over 15 min was recorded.^26^ The calculation of the inhibition percentage is as follows:

$$\%\text{Inhibition}=\frac{(W_{C}-W_{T)}}{W_{C}}\times 100$$

In this context, *W*_C_ and *W*_T_ denote the mean number of writhing responses observed in the control and the test groups, respectively.

#### 2.7.2 Formalin‐induced lick test

The mice were grouped and treated as described in Section 2.6. Thirty minutes post‐treatment, 20 μL of 2.5% formalin (diluted in saline) was injected into the ventral surface of the right paw. Pain responses were evaluated by measuring the duration of paw licking during the neurogenic phase (0–5 min) and the inflammatory phase (15–30 min). AEAL was administered at doses of 200 and 400 mg/kg 1 h prior, based on a time–response curve, whereas diclofenac sodium (10 mg/kg) was used as the positive control.^27^

#### 2.7.3 Hot plate test

The mice were grouped and treated as described in Section 2.6. After treatment, the animals were placed on Eddy's hot plate to assess their nociceptive response.^28^ The hot plate temperature was maintained at 55 ± 0.5°C, with a cutoff time of 30 s to prevent injury to the mice's paws. After oral administration of the test substances, the latency to nociceptive response was assessed by recording the time at which mice exhibited a jumping response—a reflexive reaction to thermal pain—on a hot plate or similar apparatus. Measurements were taken at specific time intervals: 0, 30, 60, 90, 120, 180, and 240 min post‐administration.^29^

### 2.8 Anti‐inflammatory assay

#### 2.8.1 In vitro human red blood cell membrane stabilization assay

The anti‐inflammatory activity was assessed using the human red blood cell (HRBC) membrane stabilization method.^30^ Different concentrations of the leaf extract (1000, 500, 250, 125, and 62.5 μg/mL) and the positive control (diclofenac sodium) were prepared as described in previous studies and placed in separate test tubes. To each tube, 0.5 mL of HRBC suspension, 1 mL of phosphate buffer and 2 mL of hypo‐saline were added. The mixtures were incubated at 37°C for 30 min, then cooled and centrifuged at 4000 RPM for 20 min. The absorbance of the supernatant was subsequently measured at 560 nm using a UV spectrophotometer, with distilled water serving as the blank.

The percentage of HRBC membrane stabilization (protection) was calculated as follows:

$$\%\text{Protection}=100-\left(\frac{\text{Optical density of test sample}}{\text{Optical density of control}}\times 100\right)$$

#### 2.8.2 In vitro protein denaturation method

In vitro protein denaturation was adopted by Sakat et al.^31^ The anti‐inflammatory potential of AEAL was evaluated using the protein denaturation inhibition assay. The reaction mixture (5 mL) comprised 0.2 mL of fresh hen's egg albumin, 2.8 mL of phosphate‐buffered saline (pH 6.4), and 2 mL of the plant extract at different concentrations. A corresponding volume of double‐distilled water was used as the control. The mixtures were incubated at 37 ± 2°C for 15 min, followed by heating at 70°C for 5 min. After cooling, absorbance was recorded at 660 nm, with the vehicle serving as a blank. Diclofenac sodium (1 mg/mL) was employed as the standard drug and processed under identical conditions for absorbance measurement.^32^ The percentage inhibition of protein denaturation was calculated as follows:

$$\%\text{Inhibition}=\frac{A_{C}-A_{S}}{A_{C}}\times 100$$

Here, *A*_C_ denotes the absorbance of the control, whereas *A*_S_ represents the absorbance of the sample.

#### 2.8.3 Xylene‐induced ear edema

The mice were grouped and treated as described in Section 2.6. One hour post‐treatment, 0.01 mL of xylene was applied to the anterior and posterior surfaces of each mouse's right ear. After 1 h of exposure, the mice were euthanized, and circular sections of both treated and untreated ears were excised using a 7 mm diameter cork borer and weighed. The difference in weight between the treated and untreated ears was then calculated.^33^

### 2.9 In vitro thrombolytic study

#### 2.9.1 Streptokinase solution preparation

To the commercially available SK (1 500 000 IU, Polamin‐Werk GmbH), 5 mL of distilled water was added and thoroughly mixed. From this suspension, 100 μL (30 000 IU) was taken as the stock solution for the in vitro thrombolysis study.^34^

#### 2.9.2 Specimen for thrombolytic test

Blood (5 mL) was collected from healthy human volunteers (*n* = 10) who had not taken NSAIDs or anticoagulants in the preceding 10 days. The same ethical approval (USTMEBBC/5/01/25) was obtained for human participation, ensuring that all procedures involving human subjects were conducted in accordance with established ethical guidelines. Written informed consent was obtained from all participants prior to their involvement in the study, and their rights, privacy, and confidentiality were rigorously protected throughout the research process. A 500 μL aliquot of blood was transferred into an Eppendorf tube and incubated at 37°C for 45 min to allow for coagulation. After clot formation, the serum was separated, and the weight of each clot‐only tube was recorded to determine the exact weight of the clot. Subsequently, 100 μL of the plant extract was added to the tubes containing the clots, and the samples were incubated at 37 °C for 90 min to evaluate thrombolytic activity.^35^

$$\%\text{Clot lysis}=\frac{\text{Weight of the lysis clot}}{\text{Weight of clot before lysis}\;}\times 100$$

### 2.10 In silico study

#### 2.10.1 Protein preparation

For the analgesic, anti‐inflammatory, and thrombolytic studies, human cyclooxygenase‐2 (COX‐2, PDB: 5IKR), a COX‐2 inhibitor (PDB: 6COX), and human tissue‐type plasminogen activator (tPA, PDB: 1A5H) were obtained from the RCSB Protein Data Bank (https://www.rcsb.org/structure) in PDB format. The protein structures were prepared in Discovery Studio 2021 by removing water molecules and heteroatoms, followed by energy minimization using the steepest descent and conjugate gradient methods in Swiss‐PDB Viewer (version 4.1.0).^36^ The optimized structures were then converted to .pdbqt format with AutoDock Tools (version 1.5.6) for molecular docking studies.^37^, ^38^

#### 2.10.2 Ligand preparation

Eighteen compounds were identified after a thorough investigation of the literature. Compounds like benzofuran, 2,3‐dihydro‐, decane,2,3,5,8‐tetramethyl‐, caryophyllene, coumarin, *cis*‐.beta.‐farnesene, sesquiphellandrene, 2‐propenoic acid, 3‐(2‐hydroxyphenyl)‐, (E)‐, nonadecane, 10‐12‐pentacosadiynoic acid, 2H‐1‐benzopyran, 6,7‐dimethoxy‐2,2‐dimethyl‐, 2,3‐hexadienoic acid, 2‐methyl‐4‐phenyl‐, methyl ester, 1H‐inden‐1‐one, 5‐(1,1‐dimethylethyl)‐2,3‐ dihydro‐3,3‐, dodecane,2,6,10‐trimethyl‐, pentadecanoic acid, 14‐methyl‐, methyl ester, *n*‐hexadecanoic acid, 9,12‐octadecadienoyl chloride, (Z,Z)‐, oxirane, tetradecyl‐ and cyclobarbital.^39^ These compounds are used for in silico molecular docking. From these compounds, 17 were retrieved from PubChem in 3D SDF format, or converted from 2D to 3D SDF using Open Babel.^40^ Ligands were minimized and exported in pdbqt format using AutoDock tools for docking.^41^ To identify the top hits, the ligands were virtually screened using PyRx.^42^

#### 2.10.3 Molecular docking analysis

The selected proteins were docked with *A. lavenia* ligands using PyRx AutoDock Vina 1.2.0, which incorporates Python integration, an extended force field, and updated docking algorithms. A semiflexible docking approach was applied, keeping the proteins rigid while allowing the ligands to remain flexible. Active sites were determined based on co‐crystallized ligands, which were redocked to validate the docking protocol using root mean square deviation (RMSD) values.^43^ Molecular docking was performed using AutoGrid to define target‐specific grid boxes with a spacing of 0.357 Å: dimensions were set to *X* = 46.072 Å, *Y* = 23.0462 Å, *Z* = 32.2003 Å for the analgesic target; *X* = 27.142 Å, *Y* = 13.062 Å, *Z* = 21.1998 Å for the anti‐inflammatory target; and *X* = 42.370 Å, *Y* = 32.0342 Å, *Z* = 19.2421 Å for the thrombolytic target. Post‐docking analyses were carried out in PyMOL to visualize ligand binding poses and assess key intermolecular interactions—including hydrogen bonds, hydrophobic contacts, and other non‐covalent forces—that stabilize the ligand–receptor complexes, thereby providing structural insights into the pharmacological potential of the phytoconstituents.

#### 2.10.4 ADME/T evaluation

The ADME/T (absorption, distribution, metabolism, excretion, and toxicity) properties of the bioactive compounds in the acetone extract of AEAL were evaluated using SwissADME and pkCSM platforms, with a focus on Lipinski's “Rule of Five” to assess drug‐likeness and pharmacokinetic suitability. These computational tools predicted key parameters, including molecular weight, lipophilicity (Log P), hydrogen‐bond donors/acceptors, topological polar surface area (TPSA), gastrointestinal absorption, blood–brain barrier permeability, cytochrome P450 metabolism, and potential toxicity endpoints. The analysis helped identify which phytoconstituents comply with established criteria for oral bioavailability and favorable pharmacokinetic profiles, thereby prioritizing promising candidates for further development as therapeutic agents.^14^, ^44^

#### 2.10.5 Toxicity evaluation

The online tools admetSAR (https://lmmd.ecust.edu.cn/admetsar1/)^45^ and PROTOX II (https://tox‐new.charite.de/) were used to assess the toxicity of the phytocompounds. The safety profiles of the compounds were revealed using these techniques to predict a number of toxicological characteristics, including immunotoxicity, toxicity class, LD_50_, hepatotoxicity, cytotoxicity, carcinogenicity, and mutagenicity.

### 2.11 DFT studies

DFT calculations were performed to analyze the electronic structure and regioselectivity of the studied donor–acceptor (D–A) systems. In addition to frontier molecular orbital (FMO) analysis, global DFT‐based reactivity descriptors were employed to overcome the limitations of FMO theory in predicting regioselectivity.^46^ All calculations were carried out using the hybrid B3LYP density functional method, which provides reliable geometries and energy parameters with good computational efficiency. Full geometry optimizations were performed without symmetry constraints using the 6‐31G(d,p) basis set as implemented in the Gaussian 09 program. The optimized structures were confirmed as true minima by the absence of imaginary frequencies. Global reactivity descriptors were calculated from the frontier molecular orbital energies. The chemical hardness (*η*), which describes resistance to electron transfer, and the electronic chemical potential (*μ*), related to charge transfer ability, were evaluated using the following expressions:

η=εLUMO−εHOMO

$$\mu =\frac{\varepsilon \text{HOMO}+\varepsilon \text{LUMO}}{2}$$

These descriptors were used to rationalize the chemical reactivity and regioselectivity of the systems under study. Molecular geometries and orbitals were visualized using GaussView 5.0.9 and ChemCraft 1.6.35.^47^

### 2.12 Prediction of activity spectra for substances

The biological activities of AEAL compounds were predicted using the PASS online server,^48^ which estimates the probable activity (Pa) and inactivity (Pi) of drug‐like molecules, with SMILES structures retrieved from PubChem.^43^, ^49^

### 2.13 Statistical analysis

Data are expressed as mean ± SEM. Statistical analyses were performed using SPSS (version 25) with one‐way analysis of variance (ANOVA) followed by Dunnett's post hoc test. Significance was defined as *p < 0.05, **p < 0.01, and ***p < 0.001. Graphs were made using MS Excel 2024.

## 3 RESULTS

### 3.1 Acute toxicity study

After oral administration of AEAL extract at doses of 100, 500, 1000, 2000, and 4000 mg/kg, no adverse behavioral changes, signs of morbidity, or mortality were observed in the treated animals during the 8‐h acute observation period and over the subsequent 14‐day monitoring period.

### 3.2 Analgesic activity

#### 3.2.1 Acetic acid‐induced writhing method

The analgesic activity of the AEAL was evaluated using the acetic acid‐induced writhing test in mice. The extract significantly reduced the number of writhes in a dose‐dependent manner. At the highest dose (400 mg/kg), AEAL produced a marked reduction in writhing episodes, with only 10.33 ± 0.67 writhes recorded compared to 22.33 ± 1.76 in the control group (*p* < 0.01), representing 53.74% inhibition of the pain response and indicating potent analgesic activity. In comparison, the standard drug diclofenac sodium (10 mg/kg) exhibited strong analgesic effects, reducing writhing to 9.67 ± 0.88 (*p* < 0.01) with a higher percentage inhibition (Table 1). These results demonstrate that AEAL possesses significant dose‐dependent analgesic properties, though to a lesser extent than those of the reference drug.

**TABLE 1:** Effects of AEAL on the acetic acid‐treated mice.

Treatment	Writhing count	% Inhibition
Control	22.33 ± 1.76	–
Diclofenac	9.67 ± 0.88**	56.70
AEAL 200	15.67 ± 0.88*	29.83
AEAL 400	10.33 ± 0.67**	53.74

*Note*: All values are shown as Mean ± SEM, and statistical analysis is done using One‐Way Analysis of Variance (ANOVA). Subsequently, *n* = 5 is employed for Dunnett's multiple comparison test, with *p < 0.05 and **p < 0.01 in comparison to the control group.

Abbreviation: AEAL, acetone extract of *A*. *lavenia* leaves.

#### 3.2.2 Formalin‐induced paw licking test

The formalin‐induced paw licking test demonstrated that the AEAL exerts dose‐dependent antinociceptive effects in both phases of pain response. In the early (neurogenic) phase, AEAL at 400 mg/kg significantly reduced pain by 57.75% (*p* < 0.05) compared to the control group (Table 2). In the late (inflammatory) phase, both 200 and 400 mg/kg doses produced significant analgesic activity (*p* < 0.01), indicating a robust anti‐inflammatory component. For comparison, the standard drug diclofenac sodium (10 mg/kg) suppressed pain by 70.43% (early phase) and 72.59% (late phase). These findings confirm that AEAL possesses dual‐modality analgesic properties, effectively mitigating both acute neurogenic and chronic inflammatory pain, with enhanced efficacy at higher doses—supporting its traditional use in pain management.

**TABLE 2:** Effects of AEAL on the formalin‐treated mice.

Treatment	Early phase		Late phase	
	Licking time (s)	% Inhibition	Licking time (s)	% Inhibition
Control	23.67 ± 1.76	–	24.33 ± 1.45	–
Diclofenac	7 ± 0.58**	70.43	6.67 ± 0.67**	72.59
AEAL 200	13.33 ± 1.45*	43.68	9.67 ± 0.33**	60.25
AEAL 400	10 ± 1.53*	57.75	8.33 ± 0.33**	65.76

*Note*: All values are shown as Mean ± SEM, and statistical analysis is done using One‐Way Analysis of Variance (ANOVA). Subsequently, *n* = 5 is employed for Dunnett's multiple comparison test, with *p < 0.05 and **p < 0.01 in comparison to the control group.

Abbreviation: AEAL, acetone extract of *A*. *lavenia* leaves.

#### 3.2.3 Hot plate test

**FIGURE 1:**
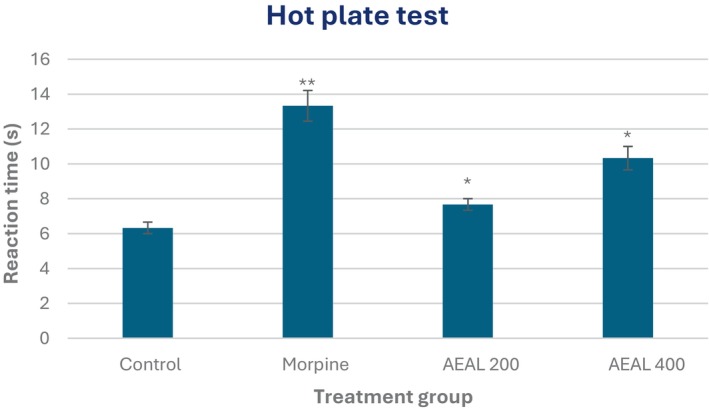
Evaluation of analgesic activity of the acetone extract of *A. lavenia* leaves (AEAL) through the hot plate test. All values are shown as Mean ± SEM, and statistical analysis is done using One‐Way Analysis of Variance (ANOVA). Subsequently, *n* = 5 is employed for Dunnett's multiple comparison test, with *p < 0.05 and **p < 0.01 in comparison to the control group." with the current caption.

The analgesic activity of the AEAL extract was further evaluated using the hot plate test, which assesses central analgesic mechanisms. Administration of AEAL at 400 mg/kg significantly increased reaction latency time in jumping from the hot plate (*p* < 0.05) compared to the control group (Figure 1), indicating enhanced pain tolerance mediated through central nervous system pathways. The standard drug morphine (10 mg/kg) showed a pronounced effect, with a reaction time of 13.33 ± 0.88 s (*p* < 0.01) compared to 6.33 ± 0.33 s in the control group, reflecting strong centrally mediated analgesia. These results suggest that AEAL exerts significant central analgesic activity, particularly at the higher dose, although its effect was less pronounced than that of morphine.

### 3.3 Anti‐inflammatory activity

#### 3.3.1 Xylene‐induced ear edema

The anti‐inflammatory activity of the AEAL was evaluated using the xylene‐induced ear edema model in mice. AEAL significantly reduced ear edema in a dose‐dependent manner compared to the control group. At a dose of 200 mg/kg, AEAL produced an ear weight difference of 1.28 ± 0.14 mg (*p* < 0.05), corresponding to 36.95% inhibition, whereas the higher dose (400 mg/kg) resulted in a more pronounced effect, with an ear weight difference of 0.91 ± 0.26 mg (*p* < 0.001) and 55.17% inhibition. These results indicate that AEAL exerts dose‐dependent anti‐inflammatory activity, with increasing efficacy at higher doses. In comparison, the reference drug indomethacin (10 mg/kg) exhibited stronger inhibition, achieving a 63.55% reduction in edema (Table 3). The observed anti‐inflammatory effect of AEAL suggests its potential as a natural agent for the management of acute inflammation.

**TABLE 3:** Effects of AEAL on the xylene‐treated mice.

Treatment	Ear weight difference (mg)	% Inhibition
Control	2.03 ± 1.58	–
Indomethacin	0.74 ± 0.44***	63.55
AEAL 200	1.28 ± 0.14*	36.95
AEAL 400	0.91 ± 0.26***	55.17

*Note*: All values are shown as Mean ± SEM, and statistical analysis is done using One‐Way Analysis of Variance (ANOVA). Subsequently, *n* = 5 is employed for Dunnett's multiple comparison test, with *p < 0.05 and ***p < 0.001 in comparison to the control group.

Abbreviation: AEAL, acetone extract of *A*. *lavenia*.

#### 3.3.2 HRBC membrane stabilization assay

**FIGURE 2:**
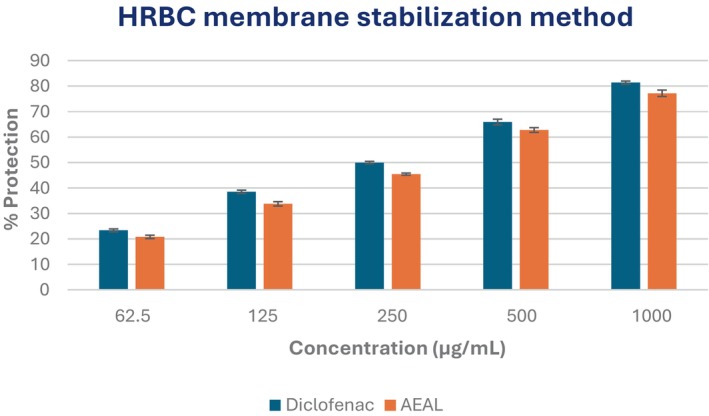
Evaluation of anti‐inflammatory activity of the acetone extract of *A. lavenia* leaves (AEAL) through the human red blood cell (HRBC) membrane stabilization assay.

**FIGURE 3:**
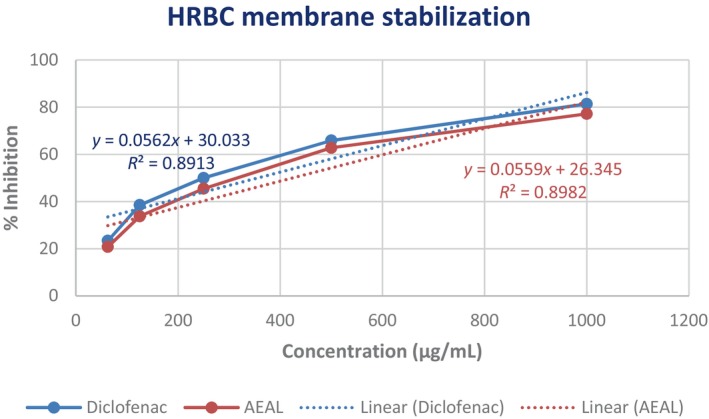
IC_50_ calculation of acetone extract of *A. lavenia* leaves (AEAL).

The anti‐inflammatory potential of the AEAL extract was further evaluated using the heat‐induced red blood cell (HRBC) membrane stabilization assay, which serves as an in vitro model for assessing membrane‐stabilizing and anti‐inflammatory activity. Both AEAL and the reference drug diclofenac sodium exhibited a dose‐dependent protective effect on the erythrocyte membrane. At the highest tested concentration of 1000 μg/mL, AEAL demonstrated a maximum protection of 77.19%, indicating significant membrane stabilization. In comparison, diclofenac provided slightly higher protection, with 81.39% inhibition of hemolysis at the same concentration (Figure 2). The IC_50_ values for diclofenac and AEAL were 355.28 and 423.17 μg/mL, respectively (Figure 3). These results suggest that AEAL stabilizes lysosomal membranes—a key mechanism in mitigating inflammatory responses—supporting its potential as a natural anti‐inflammatory agent.

#### 3.3.3 Protein denaturation assay

**FIGURE 4:**
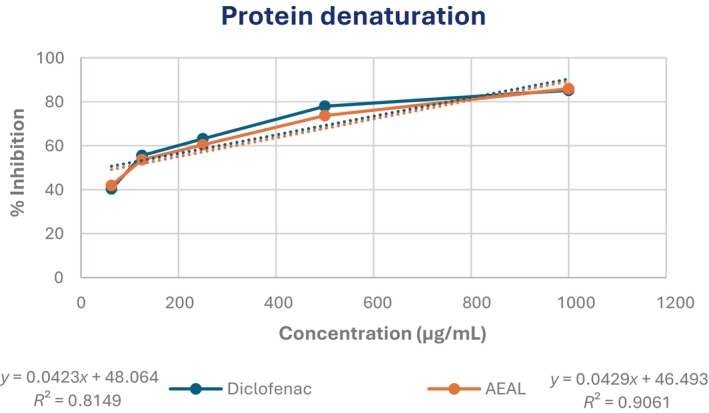
Evaluation of anti‐inflammatory activity of the acetone extract of *A. lavenia* leaves (AEAL) through the protein denaturation method.

The AEAL extract demonstrated significant anti‐inflammatory activity in the in vitro protein denaturation assay, a commonly used method to evaluate the anti‐arthritic and anti‐inflammatory potential of bioactive compounds. The extract exhibited a concentration‐dependent inhibition of protein denaturation, with a maximum inhibition of 85.96% at 1000 μg/mL. The IC_50_ value was determined to be 81.75 μg/mL, indicating potent activity. In comparison, the standard drug, diclofenac sodium, showed greater potency, with an IC_50_ of 45.77 μg/mL. These results, presented in Figure 4, suggest that AEAL has notable anti‐inflammatory properties.

### 3.4 Thrombolytic activity

#### 3.4.1 Blood clot lysis method

**FIGURE 5:**
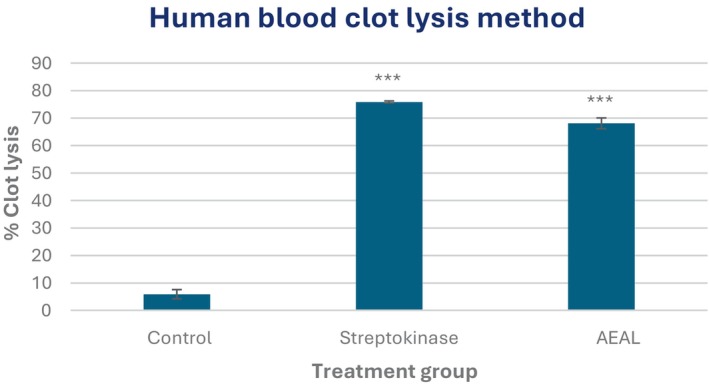
Evaluation of thrombolytic activity of the acetone extract of *A. lavenia* leaves (AEAL) through the human blood clot lysis method. All values are shown as Mean ± SEM, and statistical analysis is done using One‐Way Analysis of Variance (ANOVA). Subsequently, *n* = 5 is employed for Dunnett's multiple comparison test, with ***p < 0.001 in comparison to the control group.

The AEAL extract demonstrated significant thrombolytic potential, with 68.1% ± 1.99% clot lysis observed, compared to only 5.88% ± 1.67% in the control (*p* < 0.001). The standard thrombolytic agent streptokinase also exhibited potent (*p* < 0.001) clot‐dissolving activity (75.86% ± 0.36%), serving as a positive control (Figure 5).

### 3.5 In silico study

#### 3.5.1 ADME and drug‐likeness analysis

The pharmacokinetic properties of the identified phytochemicals from *A. lavenia* are summarized in Table 4. These results were obtained through in silico ADMET predictions using the pkCSM and SwissADME platforms, as well as drug‐likeness assessments via admetSAR. The analyses revealed favorable pharmacokinetic profiles for several compounds, indicating good oral bioavailability, membrane permeability, and metabolic stability, with low toxicity risks—key attributes for potential drug candidates. Toxicity profiles of *A. lavenia* compounds are presented in Table 5.

**TABLE 4:** ADME and drug‐likeness properties of AEAL compounds.

Compound name	Lipinski rules				Lipinski's violation ≤1	Veber's rules		Human intestinal absorption	Human oral bioavailability	Blood brain barrier (BBB)
	MW (g/mol) < 500	HBA <10	HBD <5	Log *p* ≤5		n RB ≤10	TPSA ≤140 (Å^2^)			
Decane,2,3,5,8‐tetramethyl‐	198.39	0	0	3.86	1	7	0.00	94.101	0.55	0.855
Caryophyllene	204.35	0	0	3.25	1	0	0.00	94.845	0.55	0.733
Coumarin <3,4‐dihydro‐>	146.14	2	0	1.75	0	0	30.21	97.344	0.55	−0.007
*cis*‐.beta.‐Farnesene	204.35	0	0	3.86	1	7	0.00	93.432	0.55	0.835
Sesquiphellandrene	204.35	0	0	3.58	1	7	0.00	94.668	0.55	0.787
2‐Propenoic acid, 3‐(2‐hydroxyphenyl)‐, (E)‐	164.16	3	2	1.09	0	2	57.53	93.494	0.85	−0.225
Nonadecane	268.52	0	0	5.46	1	16	0.00	90.015	0.55	0.996
10‐12‐Pentacosadiynoic acid	374.60	2	1	5.51	1	18	37.30	92.506	0.85	−0.36
2H‐1‐Benzopyran, 6,7‐dimethoxy‐2,2‐ dimethyl‐	220.26	3	0	2.96	0	2	27.69	92.503	0.55	−1.332
2,3‐Hexadienoic acid, 2‐methyl‐4‐phenyl‐, methyl ester	216.28	2	0	3.07	0	16	26.30	96.762	0.55	0.287
1H‐Inden‐1‐one, 5‐(1,1‐dimethylethyl)‐2,3‐ dihydro‐3,3‐	216.32	1	0	2.87	0	1	17.07	95.901	0.55	0.429
Dodecane,2,6,10‐trimethyl‐	212.41	0	0	4.20	1	9	0.00	93.737	0.55	0.891
Pentadecanoic acid, 14‐methyl‐, methyl ester 21 205	270.45	2	0	4.59	1	14	26.30	92.48	0.55	92.48
*n*‐Hexadecanoic acid	256.42	2	1	3.85	1	14	37.30	92.004	0.85	−0.111
9,12‐Octadecadienoyl chloride, (Z,Z)‐	298.89	1	0	3.85	1	14	17.07	91.195	0.85	0.81
Oxirane, tetradecyl‐	240.42	1	0	4.46	0	13	12.53	92.749	0.55	0.868
Cyclobarbital	236.27	3	2	1.45	0	2	75.27	80.555	0.55	0.052

**TABLE 5:** Toxicity profiles of *A. lavenia* compounds.

Compound name	Predicted LD50 (mg/kg)	Predicted toxicity class	AMES toxicity	Hepato‐toxicity	Carcinogenicity	Mutagenicity	Immunotoxicity	Cytotoxicity
Decane,2,3,5,8‐tetramethyl‐	750	3	−	−	−	−	−	−
Caryophyllene	5300	5	−	−	−	−	+	−
Coumarin	196	3	−	−	+	−	−	+
*cis*‐.beta.‐Farnesene	5000	5	−	−	−	−	−	−
Sesquiphellandrene	5000	5	−	−	−	−	−	−
2‐Propenoic acid, 3‐(2‐hydroxyphenyl)‐, (E)‐	2850	5	−	−	+	−	−	−
Nonadecane	750	3	−	+	−	−	−	−
10‐12‐Pentacosadiynoic acid	250	3	+	−	−	−	−	−
2H‐1‐Benzopyran, 6,7‐dimethoxy‐2,2‐ dimethyl‐	500	4	−	−	+	+	−	−
2,3‐Hexadienoic acid, 2‐methyl‐4‐phenyl‐, methyl ester	5000	5	−	−	−	−	−	−
1H‐Inden‐1‐one, 5‐(1,1‐dimethylethyl)‐2,3‐ dihydro‐3,3‐	1700	4	−	−	−	−	−	−
Dodecane,2,6,10‐trimethyl‐	750	3	−	−	−	−	−	−
Pentadecanoic acid, 14‐methyl‐, methyl ester 21 205	5000	5	−	−	−	−	−	−
*n*‐Hexadecanoic acid	900	4	−	−	−	−	−	−
9,12‐Octadecadienoyl chloride, (Z,Z)‐	5000	5	−	−	−	+	−	−
Oxirane, tetradecyl‐	500	5	−	−	+	−	−	−
Cyclobarbital	840	4	−	−	+	−	−	−

*Note*: Presence (+), absence (−).

#### 3.5.2 Molecular docking study

**FIGURE 6:**
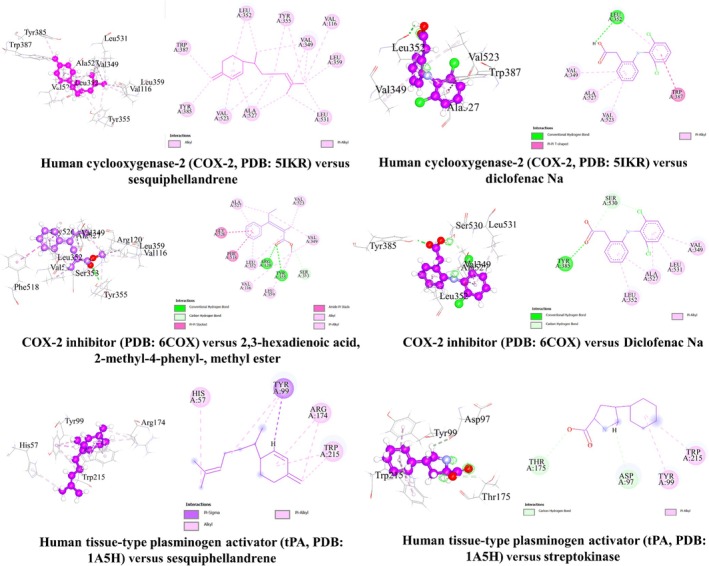
Two‐ and three‐dimensional representations of top docked compounds from the acetone extract of *A. lavenia* against important drug targets.

The combined docking scores of the selected compounds are detailed in Table 6. The docking scores and interaction analyses of the top compound from *A. lavenia* for each targeted biological activity, along with the corresponding reference drugs docked against the same protein targets, are presented in Table 7 and illustrated in Figure 6. These results highlight the binding affinities, molecular interactions, and potential binding modes of the compounds within the active sites of the target proteins, providing insights into their mechanism of action and supporting their potential as lead candidates for further drug development.

**TABLE 6:** Docking scores of the AEAL substances.

Compounds	PubChem CID	Docking score (kcal/mol)		
		Analgesic (6COX)	Anti‐inflammatory (5IKR)	Thrombolytic (1A5H)
Decane,2,3,5,8‐tetramethyl‐	545611	−6.5	−6.2	−5.4
Caryophyllene	5281515	−5.7	−6.9	−6.6
Coumarin	323	−7	−6.9	−6.3
*cis*‐.beta.‐Farnesene	5317319	−6.7	−6.8	−5.5
Sesquiphellandrene	519764	**−7.7**	−7.3	**−6.9**
2‐Propenoic acid, 3‐(2‐hydroxyphenyl)‐, (E)‐	637540	−6.8	−6.7	−6.3
Nonadecane	12401	−6.4	−6.2	−5.4
10‐12‐Pentacosadiynoic acid	538433	−6.5	−6.6	−5.6
2H‐1‐Benzopyran, 6,7‐dimethoxy‐2,2‐ dimethyl‐	12565	−6.8	−7	−5.4
2,3‐Hexadienoic acid, 2‐methyl‐4‐phenyl‐, methyl ester	608576	−7.2	**−7.4**	−6
1H‐Inden‐1‐one, 5‐(1,1‐dimethylethyl)‐2,3‐ dihydro‐3,3‐	608582	−7.3	−6.6	−6.2
Dodecane,2,6,10‐trimethyl‐	19773	−6.6	−6.5	−5
Pentadecanoic acid, 14‐methyl‐, methyl ester 21205	21205	−6.5	−6.5	−5.7
n‐Hexadecanoic acid	985	−6.3	−6.5	−5.7
9,12‐Octadecadienoyl chloride, (Z,Z)‐	9817754	−6.8	−6.6	−6.4
Oxirane, tetradecyl‐	23741	−6.2	−6.1	−5.1
Cyclobarbital	5838	−7.4	−6.4	−6.2
Standard (diclofenac sodium and streptokinase)		−8.4	−8.1	−6.5

*Note*: Bold values represent the highest binding scores.

**TABLE 7:** Binding affinity and non‐binding interactions of the acetone extract of *A. lavenia* (AEAL) compounds for analgesic (PDB: 6COX), anti‐inflammatory (PDB: 5IKR), and thrombolytic (PDB: 1A5H) properties.

Section number	Compound	Receptor	Binding affinity (kcal/mol)	Bond type	Amino acids
1	Sesquiphellandrene	6 COX	−7.7	Alkyl	LEU352, VAL523, ALA527, VAL349, VAL116, LEU359, LEU531
				Pi‐alkyl	TYR355, TYR385, TRP387
	Diclofenac sodium (standard)		−8.4	Conventional hydrogen bond	LEU352
				Pi‐Pi T‐shaped	TRP387
				Pi‐alkyl	VAL349, VAL523, ALA527, LEU352
2	2,3‐Hexadienoic acid, 2‐methyl‐4‐phenyl‐, methyl ester	5IKR	−7.4	Conventional hydrogen bond	ARG120, TYR355
				Carbon hydrogen bond	SER353
				Pi‐Pi stacked	PHE518
				Amide‐Pi stacked	GLY526, ALA527
				Alkyl	ALA527, VAL349, LEU352, VAL523, VAL116, LEU359
				Pi‐alkyl	TYR355, LEU352, ALA527
	Diclofenac sodium (standard)		−8.1	Conventional hydrogen bond	TYR385
				Carbon hydrogen bond	SER530
				Pi‐alkyl	LEU352, ALA527, VAL349, LEU531
3	Sesquiphellandrene	1A5H	−6.9	Pi‐sigma	TYR99
				Alkyl	ARG174
				Pi‐alkyl	HIS57, TYR99, TRP215
	Streptokinase (standard)		−6.5	Carbon hydrogen bond	THR175, ASP97
				Pi‐alkyl	TYR99, TRP215

##### Docking study for analgesic activity

The analgesic potential of selected bioactive compounds from *A. lavenia* was evaluated through molecular docking studies against cyclooxygenase‐2 (COX‐2), a key enzyme in pain and inflammation pathways (PDB ID: 6COX). All tested compounds demonstrated favorable interactions with the COX‐2 enzyme's active site. Among them, sesquiphellandrene exhibited the highest binding affinity with a docking score of −7.7 kcal/mol. Although this was slightly lower than that of the reference drug, diclofenac sodium (−8.4 kcal/mol), the interaction profile of sesquiphellandrene closely resembled that of the standard. Detailed analysis revealed that sesquiphellandrene formed 10 hydrophobic interactions with critical amino acid residues in the COX‐2 binding pocket, including Leu352, Val523, Ala527, Val349, Val116, Leu359, Leu531, Tyr355, Tyr385, and Trp387—residues known to be essential for inhibitor binding. These interactions suggest a stable, specific binding mode within the catalytic site, mimicking the mechanism of action of diclofenac sodium. The results indicate that sesquiphellandrene may act as a potent COX‐2 inhibitor and contribute significantly to the observed analgesic activity of *A. Lavenia* (Table 7 [Section 1], Figure 6).

##### Docking study for anti‐inflammatory activity

In this study, all tested compounds showed strong binding affinity toward human COX‐2, indicating potential anti‐inflammatory activity. Molecular docking analysis revealed that 2,3‐hexadienoic acid, 2‐methyl‐4‐phenyl‐, methyl ester demonstrated the highest docking affinity of −7.4 kcal/mol against COX‐2 (PDB: 5IKR), which is comparable to the reference drug diclofenac sodium (−8.1 kcal/mol). A detailed analysis of the binding interactions showed that this compound formed 15 hydrophobic interactions with key amino acid residues in the active site, including Arg120, Tyr355, Ser353, Phe518, Gly526, Ala527 (3), Val349, Leu352 (2), Val523, Val116, Leu359, and Tyr355—many of which are critical for ligand binding and enzymatic inhibition. These interactions occurred at short intermolecular distances, indicating strong and stable binding within the catalytic pocket of COX‐2. The results suggest that the compound has high affinity for the COX‐2 active site and may act as a potent anti‐inflammatory agent by mimicking the binding mechanism of established NSAIDs (Table 7 [Section 2] and Figure 6).

##### Molecular docking for thrombolytic activity

All tested compounds demonstrated strong binding affinity for the human tissue‐type plasminogen activator (tPA), a key enzyme involved in fibrinolysis (PDB: 1A5H), suggesting potential thrombolytic activity. Notably, sesquiphellandrene exhibited the highest docking affinity of −6.9 kcal/mol, surpassing that of the conventional thrombolytic agent streptokinase (−6.2 kcal/mol) under the same computational conditions. Detailed interaction analysis revealed that sesquiphellandrene formed five hydrophobic interactions with critical active‐site residues: Tyr99 (2), Arg174, His57, and Trp215—residues known to play essential roles in tPA's catalytic function and ligand binding. These stable interactions at the molecular level indicate that sesquiphellandrene binds effectively to tPA's active site, potentially enhancing its fibrinolytic activity. The results suggest that sesquiphellandrene may serve as a promising lead compound for the development of novel thrombolytic agents (Table 7 [Section 3] and Figure 6).

### 3.6 Frontier molecular orbital evaluations

**FIGURE 7:**
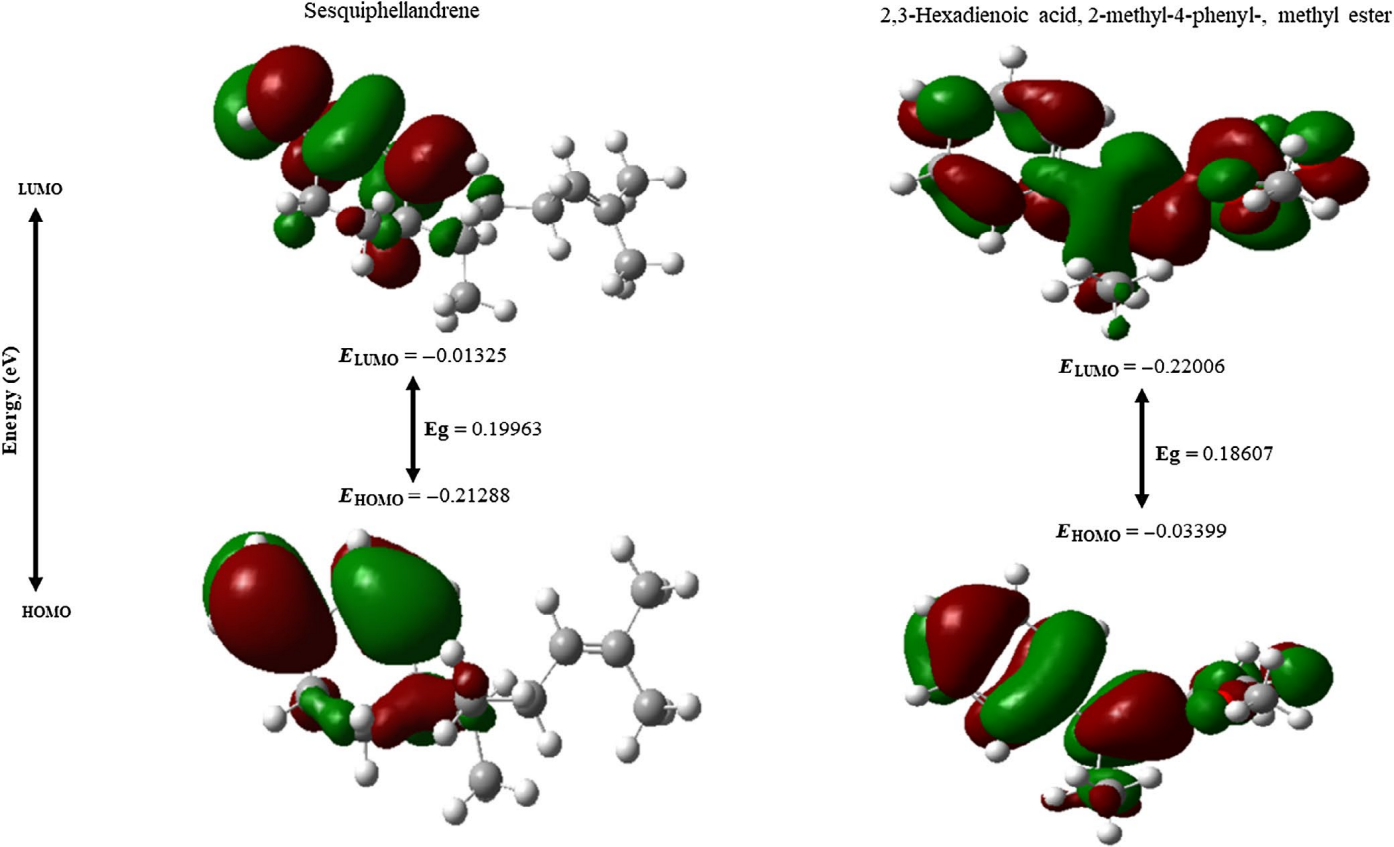
Density functional theory (DFT)‐based molecular orbital and reactivity descriptor profiling of top‐ranked compounds.

DFT was used to investigate the reactivity and drug‐likeness of sesquiphellandrene and 2,3‐hexadienoic acid, 2‐methyl‐4‐phenyl‐, methyl ester molecules. The optimized structures and HOMO and LUMO of sesquiphellandrene and 2,3‐hexadienoic acid, 2‐methyl‐4‐phenyl‐, methyl ester molecules are shown in Figure 7, along with their reactivity. Sesquiphellandrene had HOMO and LUMO energies of −0.21288 and − 0.01325 eV, with chemical hardness (*η*) of 0.09982 eV and chemical softness of 8.84447 eV^−1^, the latter of which is considerably lower, as seen in Table 8. This indicates a highly polarizable and easily reactive system, making it preferable in the initial stages of interaction with biological molecules. The distribution of HOMO‐LUMO presented in Figure 7 indicates an electron density that is highly delocalized. In addition, the values of dipole moment (1.7568 Debye) of the methyl ester compound are higher than those of sesquiphellandrene (1.0516 Debye), indicating a higher affinity for involvement in hydrogen bonds and dipole–dipole forces, with the binding sites of the receptors.

**TABLE 8:** Molecular orbital, chemical hardness, and reactivity descriptor analysis.

Properties	Sesquiphellandrene	2,3‐Hexadienoic acid, 2‐methyl‐4‐phenyl‐, methyl ester
*A* = HOMO	−0.21288	−0.22006
*I* = LUMO	−0.01325	−0.03399
*E* Gap = (*I* − *A*)	0.19963	0.18607
Chemical Potential (*μ*) = −(I + *A*)/2	0.113065	0.12703
Hardness (*ղ*) = (I − *A*)/2	0.09982	0.09304
Softness (*σ*) = 1/*μ*	8.84447	7.87216
Electronegativity (*X*) = (*I* + *A*)/2	−0.113065	−0.12703
Electrophilicity (*ω*) = *μ* ^2^/2*ղ*	0.06403	0.08672
Dipole moment (Debye)	1.0516	1.7568

Abbreviations: HOMO, highest occupied molecular orbital; LOMO, lowest occupied molecular orbital.

### 3.7 PASS prediction

Seventeen compounds isolated from *A. lavenia* were screened for potential analgesic, anti‐inflammatory, and thrombolytic activities using PASS (Prediction of Activity Spectra for Substances) analysis. The results, presented in Table 9, indicate the probability of biological activity (Pa) for each compound, highlighting several compounds with a high likelihood of the targeted pharmacological effects.

**TABLE 9:** PASS prediction of biologically active compounds of *A. lavenia*.

Compound name	Biological activity					
	Analgesic		Anti‐inflammatory		Thrombolytic	
	Pa	Pi	Pa	Pi	Pa	Pi
Decane,2,3,5,8‐tetramethyl‐	0.402	0.108	0.363	0.021	–	–
Caryophyllene	0.414	0.099	0.745	0.011	–	–
Coumarin <3,4‐dihydro‐>	0.386	0.119	0.615	0.028	0.163	0.129
*cis*‐.beta.‐Farnesene	0.388	0.118	0.326	0.139	0.491	0.003
Sesquiphellandrene	0.368	0.133	0.392	0.100	0.220	0.049
2‐Propenoic acid, 3‐(2‐hydroxyphenyl)‐, (E)‐	0.496	0.038	0.687	0.018	0.202	0.071
Nonadecane	0.565	0.012	0.424	0.084	0.280	0.014
10‐12‐Pentacosadiynoic acid	0.425	0.090	0.680	0.018	0.231	0.039
2H‐1‐Benzopyran, 6,7‐dimethoxy‐2,2‐ dimethyl‐	0.379	0.125	0.386	0.013	0.252	0.025
2,3‐Hexadienoic acid, 2‐methyl‐4‐phenyl‐, methyl ester	0.444	0.075	0.340	0.129	0.419	0.004
1H‐Inden‐1‐one, 5‐(1,1‐dimethylethyl)‐2,3‐ dihydro‐3,3‐	0.417	0.096	0.531	0.048	0.242	0.031
Dodecane,2,6,10‐trimethyl‐	0.469	0.057	0.272	0.190	0.220	0.049
Pentadecanoic acid, 14‐methyl‐, methyl ester 21205	0.490	0.042	0.392	0.100	0.299	0.009
*n*‐Hexadecanoic acid	0.526	0.023	0.515	0.052	0.326	0.006
9,12‐Octadecadienoyl chloride, (Z,Z)‐	0.501	0.036	0.382	0.028	–	–
Oxirane, tetradecyl‐	0.438	0.080	0.377	0.030	0.176	0.107
Cyclobarbital	0.374	0.129	0.290	0.094	–	–

## 4 DISCUSSION

Natural products have garnered growing interest due to their rich diversity of bioactive compounds and relatively low toxicity, making them valuable sources for drug discovery and development.^50^ The present investigation demonstrates that the AEAL possesses potent analgesic, anti‐inflammatory, and thrombolytic properties, substantiating its traditional uses.

The analgesic activity of AEAL was confirmed through multiple models. In the acetic acid‐induced writhing assay, AEAL produced significant inhibition of abdominal constrictions, with reductions of 29.83% and 53.74% at 200 and 400 mg/kg, respectively, comparable to diclofenac sodium (56.70%). This suggests that AEAL interferes with peripheral pain mechanisms, likely by inhibiting the synthesis or action of pro‐inflammatory mediators such as prostaglandins, which are key contributors to peripheral sensitization during the test. The observed effect aligns with previous reports on flavonoid‐rich plant extracts, which are known to suppress the cyclooxygenase (COX) pathway, thereby reducing prostaglandin production and other algogenic substances.^2^, ^5^ In the formalin test, AEAL significantly inhibited both the neurogenic (early) and inflammatory (late) phases of pain, with 57.75% and 65.76% inhibition at 400 mg/kg. These results indicate dual action: central modulation of nociceptive pathways in the early phase and suppression of inflammatory mediators in the late phase. Similar dual‐phase inhibition has been reported for bioactive plant extracts containing sesquiterpenes and phenolic compounds.^51^ The hot plate test results show that AEAL extract significantly increases reaction latency time at 400 mg/kg (*p* < 0.05), indicating central analgesic activity. Because this test is sensitive to centrally acting drugs,^52^ the effect suggests modulation of supraspinal pain pathways, possibly via opioid or monoaminergic systems.^53^ Indomethacin (10 mg/kg) produced a stronger effect (*p* < 0.01), as expected, confirming the model's validity. Although less potent than indomethacin, AEAL's significant analgesic effect may be due to bioactive compounds like flavonoids or alkaloids known to influence central pain mechanisms.^54^ These findings support the traditional use of AEAL in pain relief and suggest its potential as a source of centrally acting analgesics, warranting further mechanistic and phytochemical studies.

The anti‐inflammatory activity of AEAL was further substantiated through the xylene‐induced ear edema model, where it inhibited inflammation in a dose‐dependent manner. AEAL showed significant efficacy in the xylene‐induced ear edema model, with 36.95% inhibition at 200 and 55.17% at 400 mg/kg. The reference drug indomethacin outperformed AEAL with a 63.55% reduction in edema, yet AEAL still demonstrates considerable potential as a natural anti‐inflammatory agent (Table 3). This is consistent with findings from other studies indicating that phenolic compounds and flavonoids in medicinal plants play a crucial role in mitigating inflammatory responses through various mechanisms, including the inhibition of pro‐inflammatory cytokines.^55^ The HRBC membrane stabilization assay further supports AEAL's anti‐inflammatory potential, with a maximum protection of 77.19% at 1000 μg/mL. This suggests that AEAL not only stabilizes cell membranes but may also contribute to the mitigation of lysosomal enzyme release, which is a significant factor in inflammatory processes.^30^ The HRBC membrane stabilization assay further supported these results, showing 77.19% protection at 1000 μg/mL, consistent with lysosomal membrane stabilization and reduced proteolytic enzyme release. Membrane stabilization is a hallmark of anti‐inflammatory action shared by several NSAIDs and plant‐derived compounds.^30^ Additionally, AEAL inhibited protein denaturation by 85.96%, with an IC_50_ of 81.75 μg/mL, highlighting potential anti‐arthritic activity. These effects are consistent with earlier findings that flavonoids and terpenoids prevent protein denaturation and stabilize membranes, thereby attenuating inflammation.^31^

In the thrombolytic assays, AEAL exhibited impressive clot lysis at 68.1%, contrasting sharply with the control group's 5.88%. This finding suggests that AEAL may serve as a natural thrombolytic agent, a notion supported by the existing literature highlighting the fibrinolytic potential of plant‐derived compounds.^56^ The in silico docking studies reinforced these findings, revealing that sesquiphellandrene exhibited strong binding affinities for COX‐2 (−7.7 kcal/mol) and tissue‐type plasminogen activator (tPA) (−6.9 kcal/mol). Such binding affinities are indicative of the extract's capability to modulate inflammatory and thrombolytic pathways effectively. This mechanistic correlation suggests that sesquiphellandrene may directly enhance fibrinolysis, providing a molecular explanation for the in vitro clot lysis observed. Similar thrombolytic properties have been attributed to terpenes and flavonoids in other plant extracts, further supporting this hypothesis.^57^, ^58^ Thus, AEAL may provide a safer alternative to synthetic thrombolytic agents, which are associated with hemorrhagic complications and immunogenicity.^23^ This suggests the presence of phytochemicals that enhance fibrinolysis, possibly by interacting with plasminogen activators. Phenolic compounds and terpenoids have previously been implicated in clot lysis activity by promoting plasmin activation or inhibiting fibrin cross‐linking.^57^, ^58^ In silico docking studies have provided valuable insights into the mechanisms behind the pharmacological effects observed in vivo. Notably, sesquiphellandrene, one of the primary bioactive compounds identified in AEAL, exhibited a strong binding affinity for cyclooxygenase‐2 (COX‐2) at −7.7 kcal/mol. This finding is particularly significant as COX‐2 is a well‐known target for analgesic and anti‐inflammatory drugs (Table 6). The binding interactions of sesquiphellandrene included multiple hydrophobic interactions with critical amino acid residues essential for inhibitor binding, mirroring those observed with standard drugs like diclofenac sodium (−8.4 kcal/mol). This suggests that sesquiphellandrene may act as a COX‐2 inhibitor, contributing significantly to the observed analgesic and anti‐inflammatory activities of AEAL. Furthermore, the in silico analysis of 2,3‐hexadienoic acid, 2‐methyl‐4‐phenyl‐, methyl ester revealed a docking score of −7.4 kcal/mol against COX‐2, indicating its potential role in the anti‐inflammatory effects of AEAL. This compound's strong binding affinity, coupled with its ability to inhibit pro‐inflammatory mediators, underscores AEAL's efficacy in managing inflammation. These computational results align with the in vivo findings, suggesting that the bioactive compounds in AEAL may work synergistically to exert their therapeutic effects through multiple pathways.

The acute toxicity study confirmed AEAL's safety, with no signs of morbidity or mortality observed up to 4000 mg/kg, placing it in the non‐toxic category according to Hodge and Sterner's classification. The favorable ADMET predictions for sesquiphellandrene and 2,3‐hexadienoic acid derivatives, including good oral bioavailability, low predicted toxicity, and compliance with Lipinski's “rule of five,” reinforce the potential of these phytochemicals as safe and drug‐like candidates for therapeutic development.^41^

FMO analysis—based on the highest occupied molecular orbital (HOMO) and lowest unoccupied molecular orbital (LUMO)—provides key insights into a molecule's electronic properties and reactivity.^59^ The HOMO–LUMO energy gap serves as a critical indicator: a smaller gap correlates with higher chemical reactivity, facilitating easier electron donation (via HOMO) and acceptance (via LUMO). Additionally, the spatial distribution of these orbitals reveals preferential sites for electrophilic/nucleophilic or radical attack—particularly within the π‐electron system—thereby aiding in the prediction of reaction sites and mechanisms in drug–target or antioxidant interactions.^60^ Based on this comparative DFT study, the methyl ester derivative exhibits superior receptor affinity and binding, making it a promising candidate molecule. In contrast, sesquiphellandrene, due to its soft/active electronic nature, could also be a potent lead molecule.

The observed pharmacological effects may be attributed to various bioactive compounds present in AEAL, including flavonoids, terpenoids, and alkaloids. Previous studies have indicated that these compounds can exert synergistic effects, enhancing their therapeutic efficacy.^61^ For instance, flavonoids have been documented to possess both analgesic and anti‐inflammatory properties through mechanisms such as COX inhibition and modulation of cytokine release.^62^, ^63^ The in silico docking studies further corroborated these potential mechanisms, highlighting the strong interactions between these compounds and their target proteins.

This study shows that AEAL exerts pharmacological effects by modulating nociception, stabilizing cell membranes, and enhancing fibrinolytic activity. Integrating in silico, in vivo, and in vitro data provides a comprehensive mechanistic understanding. However, further research is needed to isolate active compounds, conduct detailed mechanistic studies, assess chronic toxicity, and validate findings clinically. Although *A. lavenia* holds promise as a natural therapeutic resource that bridges traditional and modern medicine, these steps are essential before AEAL can be developed into standardized treatments.

## 5 CONCLUSION

The AEAL demonstrated significant analgesic, anti‐inflammatory, and thrombolytic effects across in vivo, in vitro, and in silico studies, with no signs of toxicity up to 4000 mg/kg. These results suggest that AEAL contains bioactive constituents capable of modulating pain, inflammation, and fibrinolysis, with molecular docking, DFT, and ADME/T analyses highlighting sesquiphellandrene and 2,3‐hexadienoic acid derivatives as promising lead compounds with favorable pharmacokinetic properties. Such findings not only validate the traditional use of *A. lavenia* but also provide a strong basis for its development into novel therapeutic agents. Future research should focus on bioassay‐guided isolation of active molecules, mechanistic investigations into their molecular targets, and long‐term safety evaluations.

## AUTHOR CONTRIBUTIONS

**Nusrat Jahan Moon:** Conceptualization; data curation; formal analysis; investigation; project administration; validation. **Mahathir Mohammad:** Conceptualization; investigation; methodology; project administration; resources; software; writing – original draft. **Md. Jahirul Islam Mamun:** Conceptualization; data curation; formal analysis; investigation; project administration; software; writing – original draft; writing – review and editing. **Fahmina Binty Azim Nova:** Data curation; formal analysis; methodology; resources; validation; visualization. **Nazmul Hasan Eshaque:** Investigation; methodology; resources; software; visualization; writing – original draft. **Md. Hossain Rasel:** Formal analysis; investigation; resources; software; validation; visualization; writing – original draft. **Zobayed Islam:** Methodology; resources; software; validation; visualization. **Md. Mahmudul Hasan:** Data curation; methodology; resources; software; validation; visualization. **Md. Liakot Ali:** Investigation; resources; software; validation; visualization; writing – original draft. **Md. Tanvir Chowdhury:** Data curation; formal analysis; investigation; methodology; resources; validation. **S. M. Moazzem Hossen:** Conceptualization; formal analysis; investigation; project administration; resources; supervision; writing – original draft; writing – review and editing.

## FUNDING INFORMATION

This research received no external funding.

## CONFLICT OF INTEREST STATEMENT

The authors declare no conflict of interest.

## ETHICS STATEMENT

The experimental protocols were approved (approval no.: USTMEBBC/5/01/25) by the Institutional Animal Ethics Committee of the University of Science and Technology Chittagong, Bangladesh.

## Data Availability

Data included in the article/supplementary material/referenced in the article.
